# Key Metabolic Enzymes Underlying Astrocytic Upregulation of GABAergic Plasticity

**DOI:** 10.3389/fncel.2017.00144

**Published:** 2017-05-16

**Authors:** Przemysław T. Kaczor, Jerzy W. Mozrzymas

**Affiliations:** ^1^Department of Molecular Physiology and Neurobiology, Faculty of Biological Sciences, University of WrocławWrocław, Poland; ^2^Laboratory of Neuroscience, Department of Biophysics, Wrocław Medical UniversityWrocław, Poland

**Keywords:** metabolism, inhibitory long term potentiation, miniature inhibitory synaptic currents, monocarboxylate transport, hippocampal neurons, astrocytes

## Abstract

GABAergic plasticity is recognized as a key mechanism of shaping the activity of the neuronal networks. However, its description is challenging because of numerous neuron-specific mechanisms. In particular, while essential role of glial cells in the excitatory plasticity is well established, their involvement in GABAergic plasticity only starts to emerge. To address this problem, we used two models: neuronal cell culture (NC) and astrocyte-neuronal co-culture (ANCC), where we chemically induced long-term potentiation at inhibitory synapses (iLTP). iLTP could be induced both in NC and ANCC but in ANCC its extent was larger. Importantly, this functional iLTP manifestation was accompanied by an increase in gephyrin puncta size. Furthermore, blocking astrocyte Krebs cycle with fluoroacetate (FA) in ANCC prevented enhancement of both mIPSC amplitude and gephyrin puncta size but this effect was not observed in NC, indicating a key role in neuron-astrocyte cross-talk. Blockade of monocarboxylate transport with α-Cyano-4-hydroxycinnamic acid (4CIN) abolished iLTP both in NC and ANCC and in the latter model prevented also enlargement of gephyrin puncta. Similarly, blockade of glycogen phosphorylase with BAYU6751 prevented enlargement of gephyrin puncta upon iLTP induction. Finally, block of glutamine synthetase with methionine sulfoxide (MSO) nearly abolished mIPSC increase in both NMDA stimulated cell groups but did not prevent enlargement of gephyrin puncta. In conclusion, we provide further evidence that GABAergic plasticity is strongly regulated by astrocytes and the underlying mechanisms involve key metabolic enzymes. Considering the strategic role of GABAergic interneurons, the plasticity described here indicates possible mechanism whereby metabolism regulates the network activity.

## Introduction

Phenomenon of activity-induced long-lasting potentiation of glutamatergic synaptic transmission was first unequivocally described in a seminal article by Bliss and Lømo (1973) for the perforant path to the dentate gyrus projection. Since then, activity-dependent changes in synaptic functions were described for a diversity of glutamatergic pathways revealing a number of underlying pre- and postsynaptic mechanisms (Citri and Malenka, 2008; Feldman, 2009; Huganir and Nicoll, 2013). Nowadays it is widely recognized that excitatory synaptic strength throughout the central nervous system may show temporal variations, depending on network activity patterns, the phenomenon referred to as synaptic plasticity. However, it took decades to clearly demonstrate the link between synaptic plasticity and memory formation (e.g., Whitlock et al., 2006; Neves et al., 2008; Takeuchi et al., 2014). Since glutamatergic excitatory synapses are the most numerous in the CNS, several clearly distinct long-range projections are glutamatergic and because of readily inducible plasticity phenomena, studies of glutamatergic transmission, for some time, overshadowed investigations into inhibitory GABAergic drive. However, it has been found that although GABAergic neurons and synapses are less numerous than glutamatergic ones, they play a key role in shaping the network activity, including brain rhythms (Buzsáki and Draguhn, 2004; Bonifazi et al., 2009; Jensen and Mazaheri, 2010), and related cognitive processes. The major difficulty in studying inhibitory phenomena at cellular and network level is a great heterogeneity of inhibitory interneurons with respect to e.g., their localization, morphology (Whittington and Traub, 2003; Somogyi and Klausberger, 2005; Bartos et al., 2007; Klausberger and Somogyi, 2008; Buzsáki and Wang, 2012; Roux and Buzsáki, 2015). Moreover, typically, GABAergic neurons form only local and highly specialized connections which makes it very difficult to stimulate a homogeneous subpopulation of interneurons and to record a resulting field potential (as e.g., in hippocampal CA3-CA1 projection). For these reasons, whereas glutamatergic system was regarded as a highly plastic one, GABAergic inhibition was for long perceived as a relatively static and mostly local regulator of the glutamatergic drive. However, application of tools enabling to characterize functioning of interneurons at the single cell level demonstrated that GABAergic synapses also show a bi-directional plasticity and a multitude of cell-specific pre- and postsynaptic plasticity mechanisms have been described (Rueda-Orozco et al., 2009; Castillo et al., 2011; Vogels et al., 2013; Fritschy and Panzanelli, 2014; Wang and Maffei, 2014). However, studies on molecular mechanisms of GABAergic/inhibitory plasticity mechanisms are generally hampered by the plethora of GABAergic cells and multiplicity of cell-specific mechanisms (Serrano et al., 2006; Fenselau et al., 2011; Mapelli et al., 2016; Valentinova and Mameli, 2016; Wilmes et al., 2017). Recently a model of chemically induced postsynaptic inhibitory induced long-term potentiation (iLTP) turned out to be particularly useful, enabling to indicate, for example, a crucial role of gephyrin in docking GABA_A_ receptors in the synapses and a key role of receptors lateral mobility in this type of plasticity (Petrini et al., 2014). In addition, increase in GABA_A_R number in the dendritic membrane (Marsden et al., 2007) and mechanism regulating of GABA_A_R trafficking and docking to synapse (Luscher et al., 2011; Vithlani et al., 2011; Mele et al., 2016) have been implicated in the plasticity mechanisms. Nonetheless, GABAergic plasticity mechanisms remain poorly understood and, in particular, in contrast to glutamatergic synapses, practically nothing is known about the role of astrocytes in the plasticity mechanisms. This seems interesting especially in the light of our recent studies (Kaczor et al., 2015) showing that the basal GABAergic transmission is strongly regulated by astroglial metabolism. In our recent study (Kaczor et al., 2015), we studied how modulation of GABAergic currents by astrocytes depends on key enzymes involved in cellular metabolism. In particular, we found that enrichment of the neuronal culture with astrocytes resulted in a marked increase in mIPSC frequency and this effect was accompanied by increased number of GAD65 and vGAT puncta indicating increased number of synapses. Inhibition of glutamine synthetase strongly reduced mIPSC frequency in the astrocyte-neuronal co-culture (ANCC) and a similar effect was observed when blocking glycogen phosphorylase or the astrocytic Krebs cycle. In the present study, we used neuronal culture (NC, nominally without astrocytes) and ANCC, as in Kaczor et al. (2015) in which postsynaptic iLTP was induced by a short-lasting NMDA administration, as described by Petrini et al. (2014). We report that the presence of astrocytes (in ANCC) results in a marked increase in iLTP magnitude when compared to NC. Moreover, we found that increased iLTP in ANCC is strongly dependent on astrocytic Krebs cycle and on monocarboxylate transport/signaling via respective transporters. On the other hand, inhibition of glutamine synthase appeared to interfere with iLTP both in NC and ANCC. Importantly, immunofluorescence staining of GABAergic synapses (vGAT and gephyrin, pre- and post-synaptic markers), provided results consistent with electrophysiological experiments.

## Materials and Methods

### Cell Culture

All performed procedures on rats were approved by the Dean of the Faculty of Biological Sciences at the University of Wrocław in accordance to act on animal protection used for scientific or educational purposes (issued January 15th, 2015). Cell cultures were prepared from P0–P1 Wistar rat pups using methodology described previously (Kaczor et al., 2015) with minor modifications. Briefly, pups were killed by decapitation and hippocampi were quickly removed from brains in ice cold dissociation medium, DM (in mM: 81.8 Na_2_SO_4_, 30 K_2_SO_4_, 5.8 MgCl_2_, 0.25 CaCl_2_; 1 HEPES, 20 Glucose; 1 Kynureic Acid, 0.001% Phenol Red). Dissected hippocampi were treated twice for 10 min with trypsin with Kynureic acid supplementation, rinsed three times in plating medium to block trypsin activity (MEM, 10% FBS, antibiotics) and DM. Then hippocampal tissue was mechanically dissociated with fire polished glass pipettes. Cells suspended in MEM-FBS was then transferred to 10 ml OPTI-MEM and centrifuged at 163 g. Obtained cell pellet was then re-suspended in the plating medium and plated at density 3.54 × 10^4^ cells/cm^2^ at 18 mm diameter cover slips, covered with poly-Lysine (Sigma-Aldrich, Germany) and laminin (Roche, France). To optimize observation of synaptic puncta in confocal microscopy, final cell density was set at 2.36 × 10^4^ cells/cm^2^. After 2 h, medium was changed to NA/B-27 medium (culture growth medium, Neurobasal-A w/o Phenol Red, 2% B-27 Supplement, 1% Penicillin/Streptomycin, 0.5 mM Glutamine, 12.5 μM Glutamate, 25 μM β-mercapto-ethanol). At the next day, to suppress astrocytic growth, AraC was added at a final concentration of 25 μM. Primary cell culture obtained using this protocol contained a small contamination of astrocytes (on average 10%). To prepare the ANCCs, astrocytes were centrifuged, suspended in a small volume of Hanks Balanced Salt Solution (HBSS) and added to NC at final density of approximately 4 × 10^4^ cells/cm^2^ (for electrophysiology measurements) and 2.7 × 10^4^ cells/cm^2^ for cover slips used in immunofluorescence experiments. All electrophysiology and immunofluorescence experiments were performed within 13 and 14 days of culture in the case of ANCC and not earlier than 24 h after astrocytes addition to NC (total exposure of neurons to astrocytes no less than 24 h). Astrocytes were prepared from the same animals as neurons. Cell pellet (obtained in the same way as for neurons) was suspended in astrocytic growth medium (DMEM, IITD, Poland) supplemented with 10% of fetal bovine serum (Gibco, El Paso, TX, USA), 2 mM glutamine, 5.55 mM glucose (Chempur, Poland) and 1% Penicillin/Streptomycin) and cultured in plastic 25 ml flask. To eliminate fibroblasts contamination, astrocyte growth medium was supplemented with d-Valine in final concentration (94 mg/l, 0.8 mM), to remove remaining neurons, two passages were performed, once a week before their use in the co-cultures. Unless otherwise stated, all chemicals were from Sigma-Aldrich, Germany.

### iLTP Induction

The iLTP was induced by applying the protocol used by Petrini et al. (2014) with slight modifications. Briefly, cells were transferred to ringer solution supplemented with NMDA (20 μM, Sigma-Aldrich, Germany) and CNQX (10 μM, Axon Medchem, Netherlands) for 2 min. Drugs used to block metabolic enzymes were present during the plasticity induction. Afterwards, prior recordings, coverslips were transferred for up to 20 min to recording solution (standard or containing respective drug) at 37°C for recovery.

### Tests of Pharmacological Agents on iLTP

**Figure 1:**
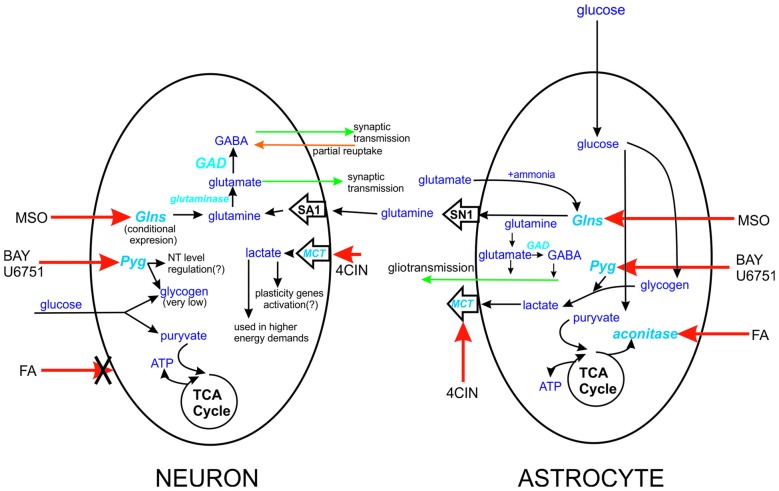
**Schematic representation of the site action of tested compounds.** Action sides of tested compounds are pointed with red arrow, aconitase-FA, monocarboxylates transporters (MCT’s)-4CIN, Pyg-BAY U6751, Glsn-methionine sulfoxide (MSO). The differences between action sides of these compounds are severe. In case of FA, we can only influence astrocytes since this agent can’t be taken by neurons. Similar but different is Glns case, expression of this enzyme in neurons is highly connected with deprivation of neurons from glutamine delivery. In case of astrocytes Glns is constitutively expressed by them and can be even considered as marker enzyme for CNS cells. Glutamine is delivered from astrocytes to neurons through SN1 and SA1 transporters, expressed in astrocytes and neurons, respectively. Factor that differentiate neuronal Pyg from its astrocytic counterpart is level of glycogen in these cells. Lastly actions of MCT’s in astrocytic-neuron cross talk. MCT’s localized in neurons are responsible for monocarboxylate compound intake, and astrocytic ones have delivery role.

The impact of the following compounds was tested on iLTP: BAYU6751 (Santa Cruz Biotech., Santa Cruz, CA, USA; blocker of glycogen phosphorylase) 5 μM, methionine sulfoxide (MSO) (blocker of glutamine synthetase) 1 mM, fluoroacetate (FA; Krebs cycle inhibitor) 10 μM and α-Cyano-4-hydroxycinnamic acid (4CIN; blocker of monocarboxylate transporters, MCT’s) 100 μM. To test each blocker, four groups of cells (both for NC and ANCC) were considered: control, cells treated by medium supplemented with respective inhibitors (BAYU6751, MSO, 4CIN and FA for 30 min), cells following iLTP induction and cells treated with respective inhibitor after induction of iLTP. As a rule, to minimize data scatter, for each considered blocker, data for each group were collected in parallel from the same culture. Thus, in this experimental protocol, iLTP was assessed for NC and ANCC for each considered drug separately. After treatment, coverslips with cells were transferred to external (recording) solution containing respective inhibitor (at the same concentration as during treatment described above) and electrophysiological recordings were carried out. To better present our approach, we provide schematic of compound action presented in Figure 1. Chemicals, unless otherwise stated, were from Sigma-Aldrich-Germany.

### Electrophysiological Recordings

mIPSC were recorded in the whole-cell configuration of the patch-clamp technique at the membrane voltage of −70 mV, using the Axopatch 200B amplifier (Molecular Devices Corporation, Sunnyvale, CA, USA). Signals were acquired at 50 kHz following low-pass filtering at 5 kHz using Digidata 1440A (Molecular Devices Corporation) interface and pClamp10 software (Molecular Devices Corporation). The intrapipette solution contained (in mM): 137 CsCl, 1 CaCl_2_, 2 MgCl_2_, 11 EGTA, 2 ATP and 10 HEPES, pH 7.2 with CsOH. The composition of the external solution was the following (in mM): 137 NaCl, 5 KCl, 2 CaCl_2_, 1 MgCl_2_, 24 glucose and 10 HEPES, pH 7.2 with NaOH. To separate GABAergic currents from other events, mIPSC were recorded in the presence of 1 μM tetrodotoxin (TTX) to suppress neuronal excitability and 10 μM CNQX to block the glutamatergic currents (mainly, those mediated by AMPA receptors). The rise time kinetics of mIPSCs was described as 10%–90% onset time and the decay time course was fitted with a sum of two exponential functions: *T*_dec_(*t*) = *A*_1_exp*τ*_1_/*t* + *A*_2_exp*τ*_2_/*t* where *τ*_1_, *τ*_2_ are the time constants and *A*_1_, *A*_2_ are respective amplitudes. Based on this fit, the weighted decay time constant was calculated: *τ*_mean_ = *a*_1_*τ*_1_ + *a*_2_*τ*_2_, where *a*_1_, *a*_2_ are normalized amplitudes (*a*_1_ = *A*_1_/(*A*_1_ + *A*_2_), *a*_2_ = *A*_2_/(*A*_1_ + *A*_2_). Detection and analysis of mIPSC time course was performed with Clampfit 10 software (Molecular Devices Corporation, Sunnyvale, CA, USA). Statistical significance of mIPSC amplitude, frequency and their kinetic were assessed using unpaired two-tailed *T*-test accompanied by Fisher and normality test. Resistance of patch pipette filled with internal solution was in the range of 4–6 MΩ. Passive membrane properties (capacitance 44–65 pF, resistance 150–250 MΩ) were monitored in each whole-cell recording and no difference in these parameters was found among the groups considered in this study (comparison with Kruskal-Wallis test). Series resistance was also measured and typically was below 10 MΩ. Recordings with series resistance higher than 15 MΩ or with current rundown larger than 20% were discarded from analysis. Differences between analyzed data sets were considered statistically significant when *p* ≤ 0.05.

### Immunofluorescence Staining

Immunofluorescence staining was performed for each of above mentioned four groups of cells (both NC and ANCC). Cells were fixed with 50:50 methanol-acetone mixture at −20°C (both from POCH, Poland) for 20 min. After fixation, cover slips with cells were washed three times with PBS at the room temperature (RT). Prior to application of the primary antibodies, to prevent non-specific reaction, fixed cells were incubated with blocking buffer containing 5% BSA and 0.3% Tween 80 in PBS for 60 min. Primary antibodies were diluted to desired concentration in antibodies dilution buffer containing 1% BSA and 0.3% of tween 80. Reactions with primary antibodies were run overnight at 4°C. Secondary antibodies were diluted in the same buffer as primary ones and applied the next day for 60 min. Cell nuclei were visualized with DAPI. After each step, fixed cells were washed with fresh PBS. Cultures snapshots at 1600 × 1600 resolution with highest possible magnitude (×600, ×60 objective), were acquired with Olympus (Japan) fluo-view 1000 confocal microscope and analyze with freeware Fiji software (Fiji is just ImageJ^1^). Each snapshot of cell culture was taken to get the best sight on single cell (if possible) with nearest dendrites. Then we focused on changes in size and fluorescence intensity of puncta stained against gephyrin that colocolized (in the range of ±2 pixels) with puncta stained against vGAT. Fluorescence intensity and size of gephyrin puncta were then expressed as arbitrary units (a.u.). Primary antibodies used and their dilution were: gephyrin polyclonal rabbit 1:1000 (Cat. No. 147 002), vGAT monoclonal mouse 1:100 (Cat. No. 131 011) both antibodies from Synaptic Systems, Germany. Primary antibodies were visualized with Alexa Fluor 488 (A-11034) and Alexa Fluor 633 (A-21052) conjugated secondary antibody, against respectively rabbit and mouse in effective dilution 1:4000 (both from Thermo Fisher Scientific, Waltham, MA, USA). Other chemicals, unless otherwise stated, were from Sigma-Aldrich, Germany. Statistical significance was assessed using unpaired two-tailed *T*-test accompanied by Fisher and normality test or Mann-Whitney *U* test when normality test failed. Differences between data sets were considered statistically significant when *p* ≤ 0.05.

## Results

### Astrocytes Enhance iLTP in Primary Hippocampal Neuronal Culture

**Figure 2:**
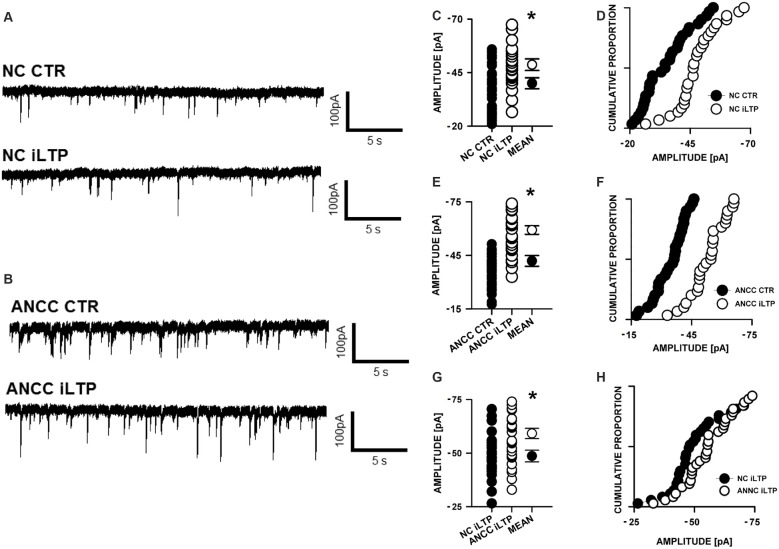
**Supplementation of neuronal cultures (NC) with astrocytes strongly upregulates inhibitory long-term potentiation (iLTP). (A)** Typical traces obtained in NC. **(B)** Typical traces from astrocyte-neuronal co-culture (ANCC), upper one present control measurement, lower one after iLTP induction. **(C,D)** Comparisons of mIPSC amplitude values obtained in NC with or without iLTP induction. **(E,F)** Comparisons of mIPSC amplitude values obtained in ANCC with or without iLTP induction. **(G,H)** Comparisons of mIPSC amplitude values obtained in NC and ANCC with iLTP induction. Statistical significant results in scatter plots (*p* < 0.05) are represented with star mark.

**Figure 3:**
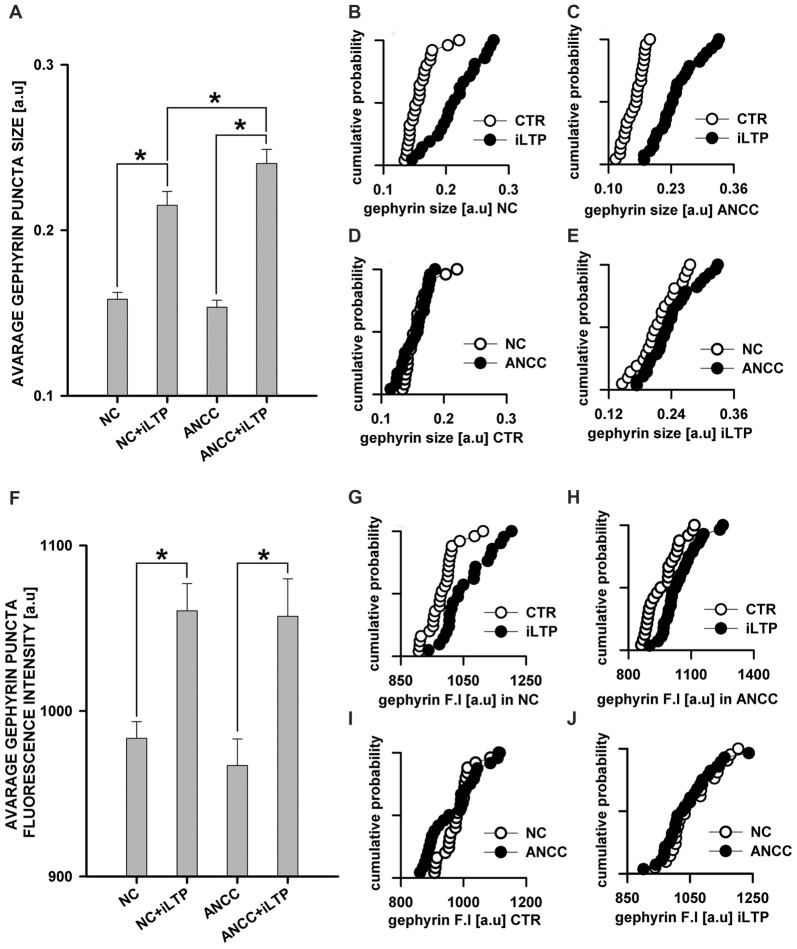
**Supplementation of NC with astrocytes strongly upregulate morphological changes introduced by iLTP. (A)** Comparison of average gephyrin puncta size in NC and ANCC with or without iLTP induction. Cumulative representation of gephyrin size in **(B)** NC with and without iLTP induction, **(C)** ANCC with and without iLTP induction, **(D)** NC and ANCC without stimulation, **(E)** NC and ANCC with iLTP induction. **(F)** Comparison of average gephyrin puncta fluorescence intensity in NC and ANCC with or without iLTP induction. Cumulative representation of gephyrin fluorescence intensity in **(G)** NC with and without iLTP induction, **(H)** ANCC with and without iLTP induction, **(I)** NC and ANCC without stimulation, **(J)** NC and ANCC with stimulation. Statistical significant results in bar charts (*p* < 0.05) are represented with star mark.

**Figure 4:**
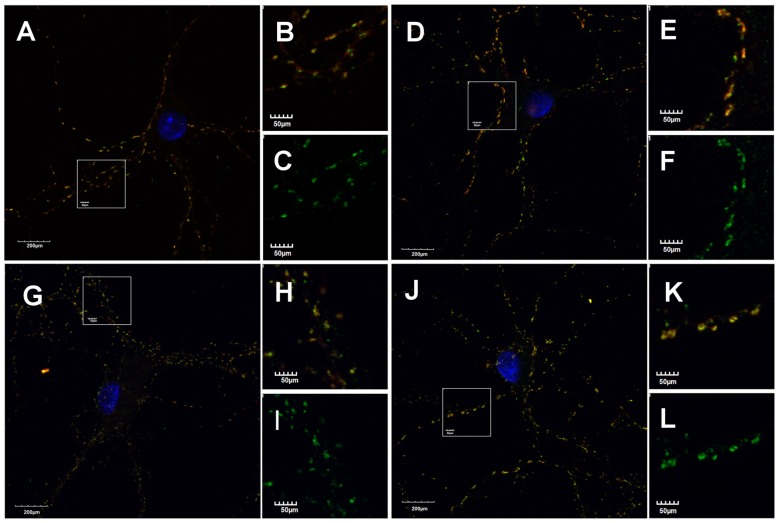
**Astrocytes affect morphology of gephyrin stained puncta.** Supplementation of NC with astrocytes increase gephyrin (green) puncta size co-stained against vGAT (red) in NC and ANCC stimulated with NMDA. **(A–C)** Typical staining’s in NC, **(D–F)** typical staining in NC stimulated with NMDA, **(G–I)** typical staining in ANCC, **(J–L)** typical staining in ANCC stimulated with NMDA. Frame squares in **(A,G,D,J)** indicate the area shown at higher magnification on the right hand side of respective pictures.

mIPSCs (Figures 2A,B) were measured in the whole-cell mode at −70 mV and in the neuronal cell culture (NC) the averaged current amplitude was −35.99 ± 1.86 pA (*n* = 31, Figures 2C,D) whereas in the ANCC −36.08 ± 1.47 pA (*n* = 34, Figures 2E,F) also rise time (1.45 ± 0.06 ms for NC and 1.53 ± 0.05 ms for ANCC) and deactivation time (30.87 ± 1.31 ms for NC and 31.45 ± 1.82 ms for ANCC) of mIPSC was unaffected and frequency was elevated in ANCC (0.17 ± 0.02 Hz for NC and 0.42 ± 0.07 Hz for ANCC). This is in agreement with our previous study (Kaczor et al., 2015) where we reported that addition of astrocytes to neuronal culture did not affect the mIPSC amplitudes or their kinetic characteristics. However, after chemically induced iLTP, we observed an increase in mIPSC amplitude both in NC and ANCC to −48.85 ± 1.96 pA (*n* = 31, *p* < 0.05, 35% increase) and −56.75 ± 1.66 pA (*n* = 34, *p* < 0.05, 57% increase), respectively (Figures 2C–H, note very clearly pronounced separation of cumulative distributions in F). Moreover, the extent of iLTP increase in ANCC was found to be significantly larger than in NC (by approximately 18%, *p* < 0.05). Chemically induced iLTP did not affect mIPSC kinetic characteristics (rise time 1.52 ± 0.04 ms for NC iLTP 1.55 ± 0.06 ms for ANCC iLTP, deactivation 29.73 ± 1.11 ms in NC and 31.29 ± 1.74 ms for ANCC) or frequency (0.16 ± 0.02 Hz for NC iLTP and 0.39 ± 0.04 Hz for ANCC iLTP). These electrophysiological data suggest that although the properties of GABA_A_Rs were not influenced by the presence of astrocytes, their number within synapses increased. To find a further support for this observation, we performed immunostainings against gephyrin, the major scaffold protein at the inhibitory synapses. To ensure that analyzed gephyrin puncta are localized synaptically, we performed a co-staining against vGAT which is a presynaptic marker of GABAergic synapses. Having identified inhibitory synapses in this way, we have determined the size and fluorescence intensity of gephyrin stained puncta. In NC, induction of iLTP resulted in 31% increase in average size of gephyrin puncta from 0.16 ± 0.004 a.u. (arbitrary units) *n* = 25 to 0.21 ± 0.008 a.u. *n* = 21, *p* < 0.05. In addition, average intensity of fluorescence was increased by approximately 8% from 983 ± 10 a.u. *n* = 25 to 1061 ± 16 a.u. *n* = 21, *p* < 0.05. Qualitatively similar results were obtained in the case of ANCC where induction of iLTP resulted in almost 50% increase of average puncta size from 0.16 ± 0.004 a.u. *n* = 24 to 0.24 ± 0.008, *n* = 28, *p* < 0.05, Figures 3A–E. Also average fluorescence intensity was increased in a similar way as in NC by approximately 9% from 967 ± 16 a.u., *n* = 24 to 1057 ± 22 a.u., *n* = 28, *p* < 0.05. Moreover, comparison of gephyrin puncta size in NC and ANCC did not reveal any significant differences. However, after iLTP induction, differences between those groups reached the statistical significance, *p* = 0.042. In the case of fluorescence intensity, comparison between NC and ANCC groups, with or without NMDA treatment, did not show any significant differences (Figures 3F–J). Examples of performed immunostaining from each group are presented in Figure 4. Altogether, these results show that the applied protocol of transient treatment with NMDA results in a larger extent of iLTP in the ANCC than in NC, and these observations are corroborated by morphologically observed enlargement of synaptic gephyrin puncta.

In our recent report (Kaczor et al., 2015), we reported that astrocytes strongly affected the basal mIPSC in a manner dependent on enzymes regulating astrocytic metabolism and therefore we checked in the present study the dependence of iLTP on these enzymes in considered models.

### Blockade of Astrocytic Krebs Cycle Differentially Affects iLTP in NC and ANCC

**Figure 5:**
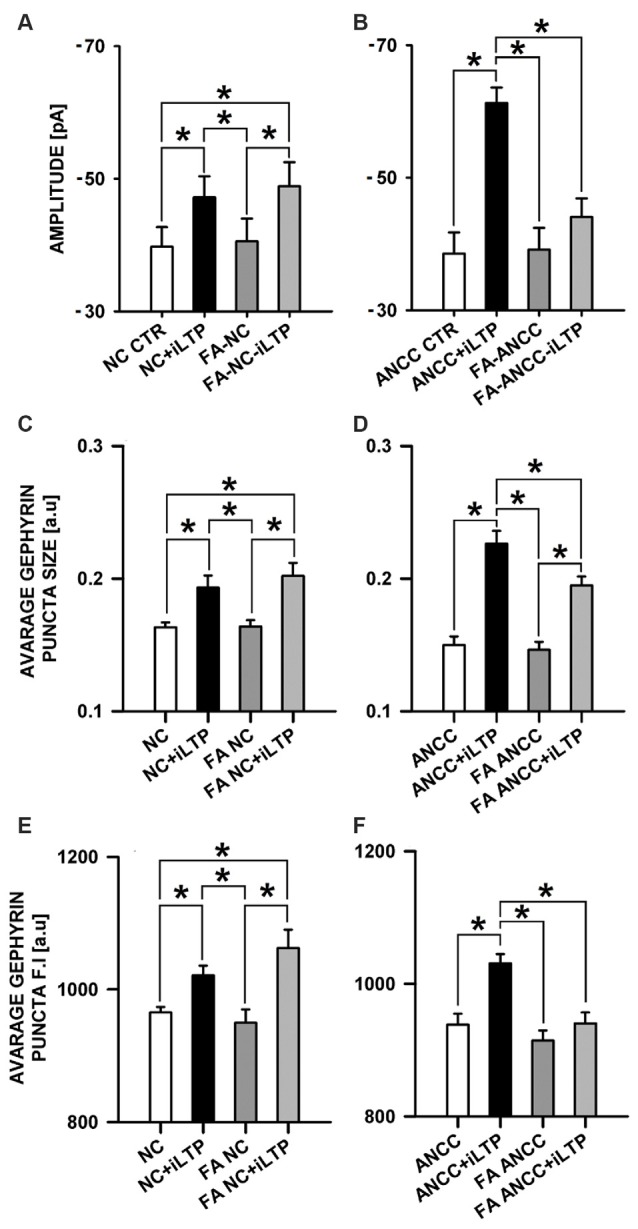
**Inhibition of astrocytic Krebs cycle by FA differentially affects iLTP in NC and ANCC.** FA abolishes astrocyte effect on iLTP in ANCC. **(A)** Average mIPSC amplitude in NC, **(B)** average mIPSC amplitude in ANCC, **(C)** average gephyrin puncta size in NC, **(D)** average gephyrin puncta size in ANCC, **(E)** average gephyrin puncta fluorescence intensity in NC, **(F)** average gephyrin puncta fluorescence intensity in ANCC. Control group (white bar), NMDA stimulated group (black bar), FA treated group (dark gray), FA treated prior to iLTP induction group (gray bar). Star mark represent statistical significant data (*p* < 0.05).

In the subsequent set of experiments we examined the impact of astrocytic Krebs cycle block by administering aconitase inhibitor, (FA, 1 μM). Treatment of NC with FA did not significantly affect the basal mIPSC amplitude (−39.75 ± 2.95 pA, *n* = 9 and −40.55 ± 3.44 pA, *n* = 9 for control and FA treatment, respectively *p* > 0.05) and the same pattern was observed for ANCC (−38.55 ± 3.18 pA, *n* = 14 and −39.14 ± 3.28 pA, *n* = 11, for control and FA treatment, respectively, *p* > 0.05). In NC, iLTP induction resulted in a marked and significant (*p* < 0.05) increase in mIPSC amplitude, for both in control (NC CTR −39.75 ± 2.95 pA *n* = 9, NC+iLTP −47.20 ± 3.17 pA, *n* = 9) and FA-treated group (FA-NC −40.55 ± 3.40 pA, *n* = 9, FA-NC+iLTP −48.86 ± 3.62 pA *n* = 9, Figure 5A). Interestingly, qualitatively different effects of FA treatment were observed in ANCC. In control conditions, iLTP induction resulted in a marked increase in mIPSC amplitude (−38.45 ± 3.18 pA, *n* = 14 and −61.27 ± 2.33 pA, *n* = 10, for baseline and iLTP, respectively, *p* < 0.05). ANCC treatment with FA largely abolished iLTP (FA-ANCC −39.14 ± 3.28 pA, *n* = 11 and FA-ANCC+iLTP −44.06 ± 2.81 pA, *n* = 14, *p* > 0.05, Figure 5B). Treatment with FA resulted in significant reduction of mIPSC frequency but this effect was limited to ANCC (0.43 ± 0.05 Hz in ANCC, 0.09 ± 0.06 Hz with FA treatment and 0.39 ± 0.06 Hz in ANCC iLTP and 0.13 ± 0.02 Hz with treatment with FA prior to iLTP inuction). Kinetic characteristic of mIPSC like rise time were unaffected by FA in all four groups that undergo treatment with FA (1.55 ± 0.05 in NC, 1.52 ± 0.05 in FA NC, 1.56 ± 0.04 in FA NC iLTP, 1.53 ± 0.05 in ANCC, 1.52 ± 0.14 in FA ANCC, 1.53 ± 0.06 in FA ANCC iLTP). However, deactivation time, was influenced by FA in a way similar to that we reported in our previous study (Kaczor et al., 2015) but this effect seemed to be unrelated to iLTP induction (32.40 ± 1.31 ms in NC, 27.04 ± 1.75 ms in FA NC, 26.07 ± 1.49 ms in FA NC iLTP, 31.32 ± 0.91 ms in ANCC, 25.44 ± 1.08 ms in FA ANCC, 27.30 ± 1.53 ms in FA ANCC iLTP). Comparison of immunostaings obtained for NC and ANCC after treatment with FA did not show any significant differences with respect to gephyrin puncta size and their fluorescence intensity. Furthermore, in the case of NC, induction of iLTP after FA treatment was fully successful and size and fluorescence intensity were increased to a similar extent as in control group (without FA treatment, Figure 5C). However, in the ANCC, we observed that FA treatment prevented iLTP and gephyrin puncta size was notably smaller than those prior iLTP induction, being for ANCC-iLTP 0.226 ± 0.0096 a.u., *n* = 21 and for FA-ANCC-iLTP 0.195 ± 0.0067 a.u., *n* = 21, *p* = 0.069 (Figure 5D). Treatment of ANCC with FA prior to iLTP induction not only prevent the increase in fluorescence puncta intensity but reduced it to the level found in NC (for ANCC iLTP 1031 ± 14 a.u., *n* = 18, and for FA ANCC LTP 940 ± 16 a.u., *n* = 21, *p* < 0.05, Figure 5F).

### The Effect of Monocarboxylate Transport Inhibition

**Figure 6:**
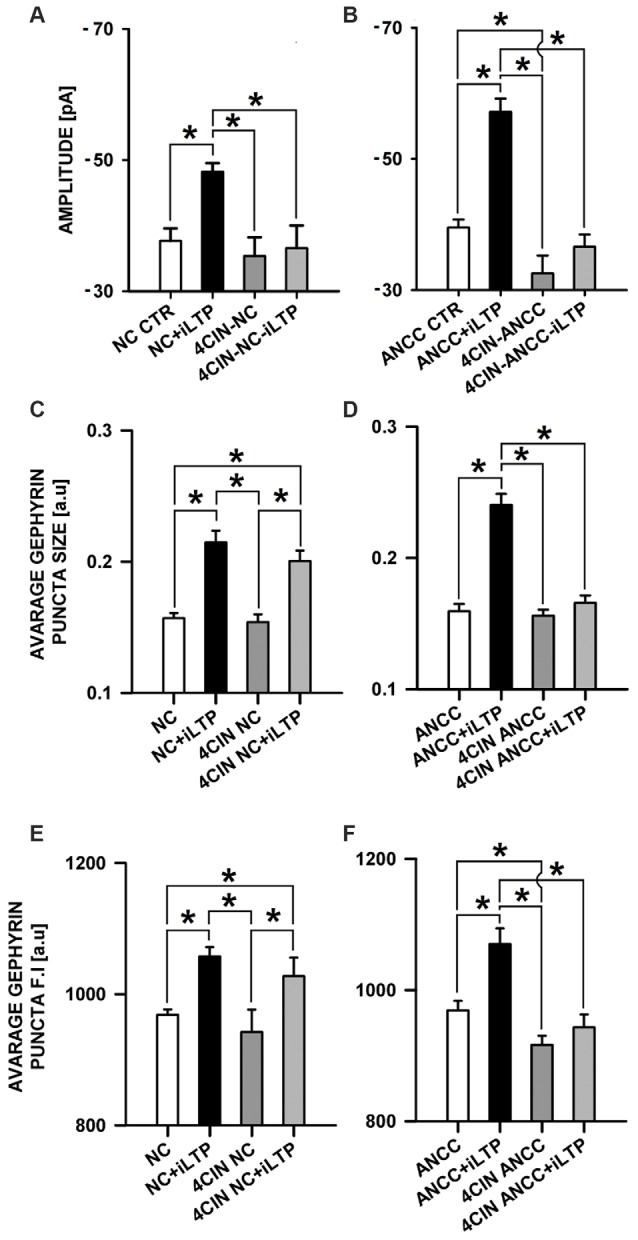
**Impact of MCT’s blockade by 4CIN.** 4CIN abolishes mIPSC amplitude increase in NC and ANCC but prevents morphological changes only in ANCC. **(A)** Average mIPSC amplitude in NC, **(B)** average mIPSC amplitude in ANCC, **(C)** average gephyrin puncta size in NC, **(D)** average gephyrin puncta size in ANCC, **(E)** average gephyrin puncta fluorescence intensity in NC, **(F)** average gephyrin puncta fluorescence intensity in ANCC. Control group (white bar), NMDA stimulated group (black bar), 4CIN treated group (dark gray), 4CIN treated prior to iLTP induction group (gray bar). Star mark represent statistical significant data (*p* < 0.05).

MCT’s play a key role in transporting the major energetic substrate (lactate) into neurons and they were found to play a critical role in glutamatergic synapse plasticity as well as in memory and learning (Suzuki et al., 2011). We decided thus to check their impact on iLTP in the considered groups. MCT’s were blocked with 100 μM 4CIN as described in “Materials and Methods” Section. In the NC, treatment with 4CIN did not significantly affect the amplitude of basal mIPSCs but prevented iLTP induction (Figure 6A). In control conditions, basal mIPSC amplitude was −37.66 ± 1.92 pA, *n* = 10 (for NC CTR) and after iLTP induction (NC+iLTP), −48.21 ± 1.29 pA, (*n* = 7, *p* < 0.05). Upon 4CIN treatment basal mIPSC amplitude in 4CIN-NC group was −35.34 ± 2.88 pA (*n* = 10) and after iLTP induction protocol, mIPSC amplitude did not change significantly (4CIN-NC+iLTP −36.56 ± 3.46 pA *n* = 10, *p* > 0.05) and was significantly smaller (*p* < 0.05) than mIPSCs amplitude recorded after iLTP induction in the control group. In the NC groups, there was no statistically significant difference between basal mIPSC amplitudes in control conditions and upon treatment with 4CIN. Interestingly, 4CIN treatment alone reduced mIPSC amplitude in ANCC in significant way (Figure 6B) contrary to the NC the (Figure 6A). In control ANCC group, iLTP induction resulted in a very pronounced and significant (*p* < 0.05) increase in mIPSC amplitude: −39.51 ± 1.23 pA, *n* = 11, vs. −57.17 ± 2.0 pA, *n* = 8 (for ANCC CTR and ANCC+iLTP respectively), following 4CIN treatment, mIPSC amplitudes were significantly smaller than in the baseline conditions in the absence of 4CIN, and iLTP protocol did not result in any significant mIPSC amplitude increase upon 4CIN treatment (−32.52 ± 2.78 pA, *n* = 10 *p* < 0.05, −37.66 ± 1.9 pA, *n* = 11, *p* > 0.05, for baseline and iLTP for ANCC upon 4CIN treatment, respectively). Values of frequency (0.17 ± 0.03 Hz in NC, 0.20 ± 0.05 Hz in 4CIN NC, 0.19 ± 0.03 Hz in 4CIN NC iLTP, 0.43 + 0.02 Hz in ANCC, 0.25 ± 0.06 Hz in 4CIN ANCC and 0.21 ± 0.05 Hz in 4CIN ANCC iLTP) mIPSC rise time (1.56 ± 0.05 ms in NC, 1.52 ± 0.05 ms in 4CIN NC, 1.58 ± 0.06 ms in 4CIN NC iLTP, 1.53 ± 0.05 ms in ANCC, 1.57 ± 0.05 ms in 4CIN ANCC, 1.54 ± 0.05 ms in 4CIN ANCC iLTP) and deactivation time (31.40 ± 0.95 ms in NC, 29.08 ± 1.35 ms in 4CIN NC, 30.82 ± 1.35 ms in 4CIN NC iLTP, 30.51 ± 0.84 ms in ANCC, 30.34 ± 1.32 ms in 4CIN ANCC, 30.13 ± 1.56 ms in 4CIN ANCC iLTP) were not influenced by 4CIN application in agreement with our previous report (Kaczor et al., 2015) These data show thus that both in NC and in ANCC blockade of MCT’s severely impairs iLTP.

**Figure 7:**
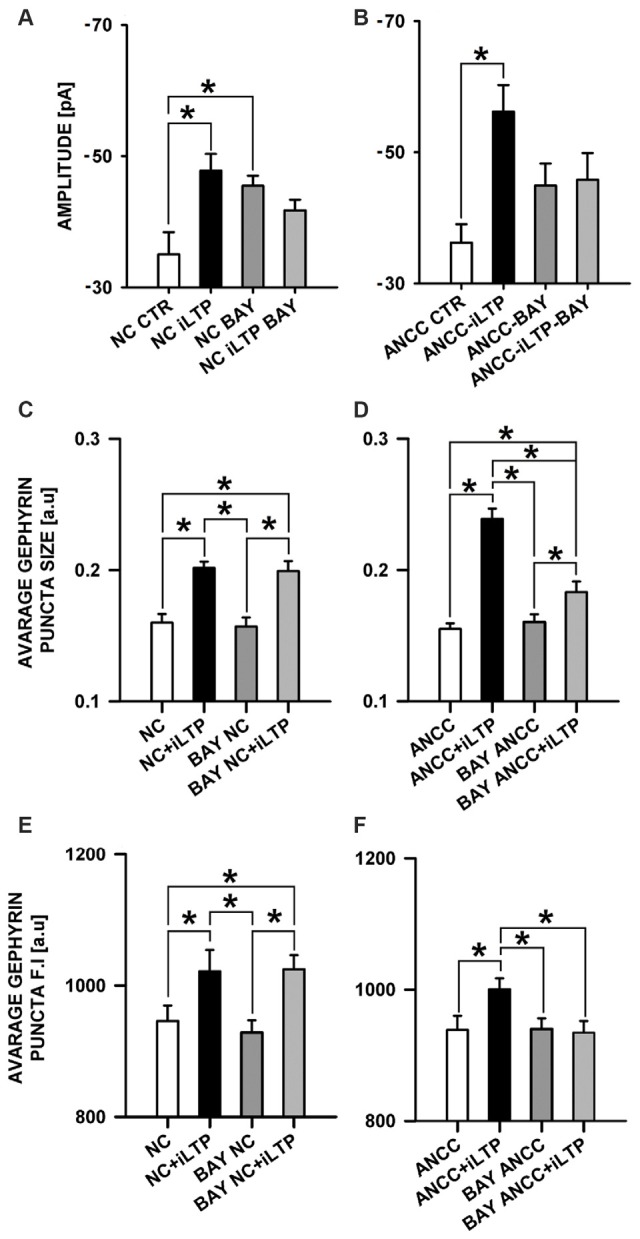
**Impact of inhibition of glycogen phosphorylase by BAY U6751.** BAY treatment prior to iLTP induction prevent morphological changes in ANCC. **(A)** Average mIPSC amplitude in NC, **(B)** average mIPSC amplitude in ANCC. Note that BAY U6751 increases mIPSC amplitudes both in NC and ANCC prior induction of iLTP. This effect renders difficult interpretation of these electrophysiological effect (see “Results” Section). **(C)** Average gephyrin puncta size in NC, **(D)** average gephyrin puncta size in ANCC, **(E)** average gephyrin puncta fluorescence intensity in NC, **(F)** average gephyrin puncta fluorescence intensity in ANCC. Control group (white bar), NMDA stimulated group (black bar), group treated with BAY (dark gray), group treated with BAY prior to iLTP induction group (gray bar). Star mark represent statistical significant data (*p* < 0.05).

Considering such a dramatic effect of monocarboxylate transport blockade on iLTP, it can be expected that inhibition of glycogen phosphorylase, which catalyzes the rate-limiting step in glycogenolysis, and thereby monocarboxylate levels, could also affect iLTP. We thus repeated the same protocol as for 4CIN for glycogen phosphorylase inhibitor (BAYU6751, 5 μM). However treatment with BAYU6751 significantly increased the basal mIPSC amplitudes in NC (−35.02 ± 3.38 pA, *n* = 12, and −45.48 ± 1.62 pA, *n* = 7, in control and BAYU6751 treated cells, *p* < 0.05, Figure 7A) and a similar effect was found in ANCC (−36.24 ± 2.8 pA, *n* = 11, vs. −44.93 ± 3.34 pA, *n* = 11, Figure 7B), however p level in the latter case reached 0.06. In addition, mIPSC amplitudes measured following iLTP induction in NC and ANCC following BAYU6751 treatment did not show any significant difference when compared to the respective baseline values (Figures 7A,B). A markedly larger mIPSC amplitude upon treatment with BAYU6751 might suggest a direct modulatory effect on GABA_A_Rs. To check this possibility we examined the impact of BAYU6751 (5 μM) on the whole-cell currents elicited by exogenous GABA applications (3 μM [GABA] applied for 4000 ms) but no effect of BAYU6751 on these currents was found arguing against such a possibility (data not shown). BAYU6751 treatment did not affected mIPSC rise time (1.53 ± 0.04 ms in NC, 1.52 ± 0.05 ms in BAY NC, 1.56 ± 0.07 ms in BAY NC iLTP, 1.55 ± 0.03 ms in ANCC, 1.57 ± 0.09 ms in BAY ANCC, 1.54 ± 0.05 ms in BAY ANCC iLTP) or deactivation time (30.95 ± 0.96 ms in NC, 30.32 ± 3.12 ms in BAY NC, 29.74 ± 1.09 ms in BAY NC iLTP, 30.62 ± 0.87 ms in ANCC, 29.67 ± 0.95 ms in BAY ANCC, 30.15 ± 1.61 ms in BAY ANCC iLTP) but mIPSC frequency (0.17 ± 0.04 Hz in NC, 0.16 ± 0.04 Hz in BAY NC, 0.15 ± 0.03 Hz in BAY NC iLTP, 0.43 + 0.02 Hz in ANCC, 0.18 ± 0.04 Hz in BAY ANCC and 0.19 ± 0.03 Hz in BAY ANCC iLTP) were affected in ANCC groups, similar to what reported in our recent study (Kaczor et al., 2015). To further extend our investigations related to the impact of MCT’s, we performed a series of immunostainings. 4CIN treatment did not change gephyrin puncta size (0.157 ± 0.004 a.u., *n* = 33 NC, 0.154 ± 0.006, *n* = 18 4CIN-NC, *p* > 0.05; Figure 5C), or its fluorescence intensity (NC 968 ± 8 a.u. *n* = 19, 4CIN-NC 942 ± 34 a.u., *n* = 15, *p* > 0.05, Figure 6E). Similar results were obtained in the case of ANCC where treatment with 4CIN did not affect gephyrin puncta size (ANCC 0.156 ± 0.005 a.u., *n* = 26, 4CIN-ANCC 0.157 ± 0.005 a.u., *n* = 24, *p* > 0.05). However, in the case of fluorescence intensity we found that 4CIN treatment resulted in its significant decrease (from 967 ± 15 a.u. *n* = 27 to 916 ± 14 a.u. *n* = 24 *p* < 0.05, Figure 5F). Moreover, 4CIN did not affect iLTP related gephyrin puncta size increase in NC (0.215 ± 0.009 a.u., *n* = 20, *p* < 0.05, Figure 6C) and their fluorescence intensity (1057 ± 14 a.u., *n* = 26, *p* < 0.05, Figure 5E). A slightly different situation we observed in ANCC where treatment with 4CIN prior to iLTP induction, prevented increase in gephyrin puncta size (iLTP 0.24 ± 0.008 a.u. *n* = 28, 4CIN+iLTP 0.166 ± 0.006 a.u., *n* = 18, *p* < 0.05, Figure 6D) and prevented also increase in fluorescence intensity (iLTP 1070.01 ± 24.00 a.u. *n* = 25, 4CIN+iLTP 943.12 ± 19.7 a.u., *n* = 18, *p* < 0.05, Figure 6F). We have additionally performed immunostainings in conditions of BAY treatment. In the case of NC, treatment with BAY did not change gephyrin size (Figure 7C) or its fluorescence intensity (Figure 7E) in the ANCC group, treatment with BAY prior to iLTP induction resulted in smaller gephyrin puncta (iLTP 0.24 ± 0.008 a.u., *n* = 26, BAY+iLTP 0.183 ± 0.008 a.u., *n* = 18, *p* < 0.05, Figure 7D) also gephyrin puncta fluorescence intensity was reduced to nearly control level (BAY+iLTP 934 ± 17 a.u., *n* = 18, control condition 938 ± 21 a.u., *n* = 21, *p* > 0.05, Figure 7F).

### The Effect of Glutamine Synthetase Inhibition

**Figure 8:**
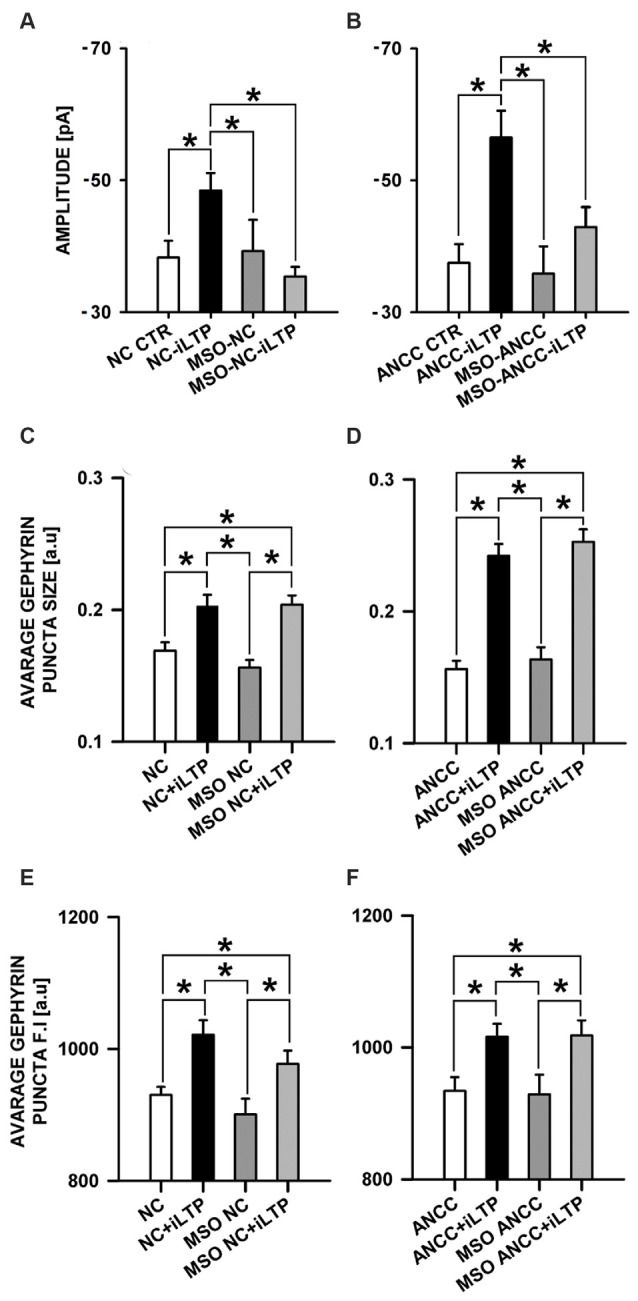
**Impact of inhibition of glutamine synthetase by MSO.** MSO treatment prior to NMDA influence mISPC amplitude changes in both NC and ANCC without inducing morphological changes. **(A)** Average mIPSC amplitude in NC, **(B)** average mIPSC amplitude in ANCC, **(C)** average gephyrin puncta size in NC, **(D)** average gephyrin puncta size in ANCC, **(E)** average gephyrin puncta fluorescence intensity in NC, **(F)** average gephyrin puncta fluorescence intensity in ANCC. Control group (white bar), NMDA stimulated group (black bar), group treated with MSO (dark gray), group treated with MSO prior to iLTP induction group (gray bar). Star mark represent statistical significant data (*p* < 0.05).

We therefore checked the impact of Glns blockade by L-MSO (1 mM, see “Materials and Methods” Section) on iLTP. Administration of MSO to NC did not affect the amplitudes of basal mIPSCs (−38.26 ± 2.56 pA, *n* = 10 and −39.24 ± 4.75 pA, *n* = 10, for controls and MSO-treated cells, respectively, *p* > 0.05, Figure 8A) and the same pattern was observed for ANCC (−37.46 ± 2.01, *n* = 10, and −35.84 ± 4.15 pA, *n* = 10 for controls and MSO-treated cells, *p* > 0.05, Figure 8B). Also in both NC and ANCC kinetic characteristic of mIPSC in MSO treatment group remain unchanged (rise time: 1.54 ± 0.06 ms in NC, 1.59 ± 0.07 ms in MSO NC, 1.59 ± 0.09 ms in MSO NC iLTP, 1.53 ± 0.06 ms in ANCC, 1.51 ± 0.09 ms in MSO ANCC, 1.56 ± 0.08 ms in MSO NC iLTP, deactivation time: 31.02 ± 1.06 ms in NC, 29.30 ± 1.35 ms in MSO NC, 30.86 ± 1.47 ms in MSO NC iLTP, 31.53 ± 1.95 ms in ANCC, 30.93 ± 1.48 ms in MSO ANCC, 30.67 ± 1.35 ms in MSO ANCC iLTP) and frequencies values (0.19 ± 0.02 Hz in NC, 0.21 ± 0.05 Hz in MSO NC, 0.19 ± 0.04 Hz in MSO NC iLTP, 0.44 ± 0.06 Hz in ANCC, 0.18 ± 0.04 Hz in MSO ANCC and 0.17 ± 0.04 Hz in MSO ANCC iTLP) showed similar ratio like in our previous report (Kaczor et al., 2015). However, NC treatment with this compound prevented iLTP (−48.42 ± 2.66 pA, *n* = 10 and 35.40 ± 1.44, *n* = 10, for mIPSC amplitudes after iLTP induction in control conditions and in the presence of MSO, respectively, *p* > 0.05). Likewise, MSO treatment of ANCC resulted in iLTP suppression (−56.48 ± 4.07 pA, *n* = 10 and −42.90 ± 3.04 pA, *n* = 10, for mIPSC amplitudes for ANCC iLTP and with MSO ANCC iLTP, respectively, *p* < 0.05). Interestingly, treatment with MSO, both NC and ANCC, did not cause any characteristic changes in gephyrin puncta size (Figures 8C,D) or its fluorescence intensity (Figures 8E,F).

## Discussion

### Regulation of iLTP by Astrocyte-Neuron Cross-Talk

The present work provides the first evidence that iLTP is enhanced by astrocytes. Indeed, although iLTP could be induced both in NC and ANCC, in the latter model, the increase in mIPSC was larger by approximately 18% (Figure 1). This result is further confirmed by morphological observations that increase in gephyrin puncta size, associated with iLTP induction, was larger in ANCC by roughly 14% (Figure 2). A clear correlation between iLTP occurrence and increase in gephyrin puncta size confirms the postsynaptic locus of GABAergic plasticity induced by the protocol used in the present study (Petrini et al., 2014). Importantly, we show that the impact of astrocytes on iLTP depends on enzymes involved in astrocyte-neuron cross-talk that have been previously shown in our laboratory to be involved in regulation of the basal GABAergic activity (Kaczor et al., 2015). Krebs cycle was blocked with FA, an inhibitor of aconitase, one of first enzyme in Krebs cycle (Cheng et al., 1972; Paulsen et al., 1987). Interestingly, FA did not impair iLTP in NC but in ANCC, FA treatment resulted in reduction of both mIPSC amplitudes and gephyrin puncta (Figure 5). These results indicate that iLTP mechanisms in NC and ANCC differ in their dependence on the Krebs cycle, revealing key role of astrocytes in utilizing the metabolic energy to develop this form of GABAergic plasticity. Surprisingly, we found that blockade of monocarboxylate transport between astrocytes and neurons with 4CIN had markedly larger impact on iLTP than inhibition of astrocytic Krebs cycle (see Figures 5, 6). Notably, whereas FA had no effect on iLTP in NC, 4CIN abolished it showing that, in contrast to our observations with FA, transport of monocarboxylates is crucial for iLTP induction both in NC and in ANCC. However, it cannot be excluded that iLTP and its dependence on MCT in NC could be, at least in part, due to some residual of astrocytes contaminating our NC. It is noteworthy that in ANCC, 4CIN not only abolished iLTP but also significantly reduced the baseline mIPSC amplitude, similar to effect described in our recent work (Kaczor et al., 2015). Reduction of mIPSC amplitudes by 4CIN has been also reported by Nagase et al. (2014) although they observed a larger effect of this compound in the case of glutamatergic synapses. Although the underlying mechanism is not clear, it is likely that, as described by Bui et al. (2004) in retina, inhibition of monocarboxylates transporters might decrease the intracellular GABA pool. Such a scenario would be consistent with our observations that in ANCC 4CIN reduces mIPSC amplitudes to the values below the baseline control, whereas postsynaptic gephyrin puncta size and intensity remain at the control level (Figure 6). Thus, in ANCC, whereas 4CIN prevents enlargement of gephyrin puncta upon iLTP induction, markedly larger effect of this compound on mIPSCs amplitude appears compatible with an additional presynaptic effect related to a reduced GABA load into the synaptic vesicles. This possibility, however, will require further experimental verification. Notably, the effect of 4CIN on the size and intensity of gephyrin puncta in NC was qualitatively different from that observed in ANCC as in the former model, iLTP induction resulted in puncta enlargement (contrary to ANCC) but no increase in mIPSC was observed. Thus, in NC a presynaptic 4CIN effect related to hypothetical decrease in the intracellular GABA pool would be a good candidate to explain this observation. To further explore this issue we have attempted to block glycogen phosphorylase (Pyg) with BAY U6751, the enzyme which is the main source for lactate and other monocarboxylates (Chih and Roberts, 2003; Hertz, 2004). However, in contrast to effect of 4CIN, inhibition of Pyg resulted in an increase in mIPSC amplitude, which was also previously reported by our group for the baseline GABAergic activity (Kaczor et al., 2015). This effect is unlikely to result from a direct modulation of GABA_A_Rs by BAY U6751 since, as mentioned in Results, this drug had no effect on current responses elicited by exogenous GABA applications. A likely explanation for this observation has been described by Sickmann et al. (2012) who found that in the type 2 diabetes model, blockade of Pyg upregulated the cellular GABA pool. However, this effect was not seen in control animals and it remains to be verified whether glycogen phosphorylase inhibition would similarly affect GABA level in our neuronal culture model. The fact that Pyg is expressed in both astrocytes and that neurons (Pfeiffer-Guglielmi et al., 2003) have an active glycogen metabolism (Saez et al., 2014), may argue in favor of this possibility. However, it needs to be considered that the amount of neuronal glycogen is by far smaller than in astrocytes (Brown and Ransom, 2007). Taken altogether, mechanisms underlying our electrophysiological observations related to Pyg inhibition are not clear as most likely they comprise several other factors involved in glycogen-dependent metabolic cascades or modulatory processes of the synaptic transmission. However, it is noteworthy that morphological data for gephyrin puncta after BAY or 4CIN treatment were very similar (Figures 5, 6). It is thus possible that postsynaptic morphological plastic changes show similar sensitivity to these two compounds because both of them ultimately affect the monocarboxylates transport between astrocytes and neurons making this process a potentially important factor in GABAergic postsynaptic plasticity. While monocarboxylates (especially lactate) are considered as the main energetic substrates for neurons (Magistretti and Pellerin, 1999; Schousboe et al., 2007; Shimizu et al., 2007) it is not clear how such a robust energy supply mechanism would result in subtle and precisely localized morphological alterations such as the observed here changes in gephyrin puncta which occur upon plasticity induction. These observations point to the novel concept of the role of monocarboxylates in astrocyte-neuron cross talk that lactate released by astrocytes, besides its energetic functions, can also be considered as an signaling molecule, especially in the processes of synaptic plasticity (Suzuki et al., 2011; Yang et al., 2014; DiNuzzo, 2016; Zhang et al., 2016).

### Glutamine-Glutamate/GABA Cycle Aspect in iLTP

We have also found that interference with glutamine-glutamate/GABA cycle by inhibiting glutamine synthetase (expressed mainly in astrocytes Martinez-Hernandez et al., 1977; Anlauf and Derouiche, 2013), with MSO markedly affected the GABAergic plasticity but this effect was different from those described for other compounds used here (FA, 4CIN and BAY). Our electrophysiological experiments showed that treatment with MSO largely prevents iLTP both in NC and ANCC, although in the latter model a small, residual iLTP was present (Figure 8). However, glutamine synthetase inhibition did not affect the morphological manifestations of iLTP—i.e., increase in size and intensity of gephyrin puncta were indistinguishable from those observed in control conditions. We can speculate that by impairing the neurotransmitter cycle, GABA level is lowered affecting presynaptic release of this neurotransmitter while postsynaptically, iLTP appears to be morphologically normal. This possibility seems contradicted, however, by a stable basal mIPSCs which would be expected to decrease upon progressive GABA depletion during the basal activity. However, GABAergic neurons were found to be very efficient in taking up GABA from the extracellular environment and thereby to maintain its activity (Yu and Hertz, 1982). It can be thus hypothesized that blockade of glutamine synthetase has its major impact in conditions of iLTP induction. It is worth mentioning in this context that several lines of evidence indicate that GABA pool and its homeostasis strongly rely on glutamine-glutamate/GABA cycle (Bak et al., 2006; Walls et al., 2015). Other scenarios of involvement of glutamine synthetase in GABAergic plasticity cannot be excluded. It is possible, for instance, that cultured hippocampal neurons, in conditions of very limited interaction with astrocytes (only ca. 10% astrocyte contamination in our NC model), are able to express glutamine synthetase, as it was shown for cerebellar granule cell by Fernandes et al. (2010). Thus neurons in NC would be forced to partially mimic the role of astrocytes in terms of synthesizing de novo neurotransmitters like GABA (Sonnewald et al., 1993).

### Astrocytic Contribution to GABAergic Plasticity

In the present study we demonstrate that astrocytes influence GABAergic plasticity through their metabolic activity, especially via monocarboxylates. However, the underlying molecular mechanisms of this regulation remain unknown. In addition to processes which are directly or indirectly regulated by metabolic cascades, a number of other potential mechanisms deserve consideration. For instance, it is known that some forms of interaction between astrocytes and neurons, that involve morphological changes of GABAergic synapses, can occur via extracellular matrix (ECM) structures such as perineuronal nets (Faissner et al., 2010), or by influencing bridging neurexin and neuroligin as it was shown for excitatory synapses (Singh et al., 2016). Involvement of these cell adhesion proteins merits attention since neurexin and neuroligin-2 co-localize in GABAergic synapses and are responsible for their formation and stabilization (Graf et al., 2004; Huang and Scheiffele, 2008; Kang et al., 2008). Another venue for studying the mechanisms of GABAergic plasticity, involved in astrocyte neuron cross-talk, is Ca^2+^ signaling which is necessary for expression of chemically induced iLTP in *in vitro* models (Petrini et al., 2014). Importantly, Ca^2+^ is involved in releasing of neurotransmitters (gliotransmitters) from astrocytes in vesicular (Domercq et al., 2006; Bergersen and Gundersen, 2009) or non-vesicular form along with other compounds that affect synaptic transmission (Takano et al., 2005; Vélez-Fort et al., 2012), pointing to gliotransmission involvement in synaptic plasticity. Our results further underscore the role of astrocytes in regulating the synaptic plasticity and provide new evidence on a crucial role of these glial cells in the plastic changes in the GABAergic drive. These observations, however, are not surprising as several previous reports clearly indicated modulatory impact of astrocytes on GABAergic drive (reviews by Araque and Perea, 2004; Losi et al., 2014). For instance Ortinski et al. (2010) have observed that GABAergic transmission is down-regulated in the presence of reactive astrocytes. Conversly, a potentiating effect of astrocytes on GABAergic inhibitory currents has been described by Christian and Huguenard (2013) who demonstrated that this up-regulating effect was due to release of endozepines from astrocytes in thalamic reticular nucleus. Wang et al. (2013) have shown that hypoosmotic stress in hypothalamic supraoptic nucleus is associated with enhancement of GABAergic currents in a manner dependent on astrocytes. This finding together with the present results indicate thus that the cross-talk between GABAergic drive and astrocyte could be a widespread phenomenon in the CNS. Astrocytes can thus exert a variety of modulatory effects on GABAergic transmission via a plethora of molecular mechanisms including e.g., numerous gliotransmitters and a rapid surge in reports describing these phenomena can be expected.

In conclusion, we report that astrocytes upregulate the GABAergic plasticity both at functional (mIPSC amplitude) and morphological (gephyrin morphology) level and provide evidence that key enzymes involved in astrocyte metabolism and astrocyte-neuron cross-talk play a key role in regulation of this plasticity.

## Author Contributions

PTK: design of the work, acquisition, analysis, interpretation of data for the work; drafting the work; final approval of the version to be published; agreement to be accountable for all aspects of the work in ensuring that questions related to the accuracy or integrity of any part of the work are appropriately investigated and resolved. JWM: substantial contributions to the conception and design of the work, interpretation of data for the work; revising it critically for important intellectual content; final approval of the version to be published; agreement to be accountable for all aspects of the work in ensuring that questions related to the accuracy or integrity of any part of the work are appropriately investigated and resolved.

## Funding

Grant sponsor: Polish National Centre of Science; grant number UMO-2015/19/N/NZ4/01023.

## Conflict of Interest Statement

The authors declare that the research was conducted in the absence of any commercial or financial relationships that could be construed as a potential conflict of interest.

## Footnotes

^1^http://fiji.sc
